# Potential of CRISPR/Cas13 System in Treatment and Diagnosis of COVID-19

**DOI:** 10.1055/s-0041-1723086

**Published:** 2021-02-25

**Authors:** Amir Khodavirdipour, Motahareh Piri, Sarvin Jabbari, Mohammad Khalaj-kondori

**Affiliations:** 1 Department of Animal Biology, Molecular Genetics Subdivision, Faculty of Natural Sciences, University of Tabriz, Tabriz, Iran; 2 Department of Anatomy, Division of Human Genetics, St. John's Hospital, Bangalore, India; 3 Department of Biology, Faculty of Sciences, University of Zabol, Zabol, Iran

**Keywords:** COVID-19, CRISPR/Cas13, diagnosis, hrsACE2, vaccines, ivermectin

## Abstract

The novel coronavirus disease 2019 (COVID-19) belongs to coronaviridae families like sarbecovirus (SARS), and causes pyrexia, pertussis, and acute respiratory distress syndrome (ARDS) in major. Started from Wuhan, China, COVID-19 now forced the World Health Organization (WHO) call it a global pandemic. These dreadful figures elevate the need for rapid action for a rapid diagnostic tool, an efficacious therapy, or vaccine for such widespread disease. In this article, we reviewed all the latest research and trials including conventional antiviral medicines that have a narrow and finite effect on COVID-19. Recently, some advances were made by a nucleotide/nucleoside analogues (NUC) inhibitor (remdesivir), ivermectin (antiparasitic drug), and convalescent plasma; the later one has more recently been approved by the Food and Drug Administration (FDA). Additionally, a clinical-grade soluble human angiotensin-converting enzyme (ACE2), named hrsACE2, was able to inhibit the infection of human blood vessel organoids, as well as the human kidney organoids, by the virus. As of now, innovative therapeutics based on the CRISPR/Cas13d might overcome the challenge of COVID-19 either as a treatment option or precise and rapid diagnostic tool due to its rapid and precise nature. In this updated comprehensive rapid review, we tried to cover all recent findings in terms of genomics, diagnosis, prevention, and treatment.

## Introduction

The first case of confirmed novel coronavirus was reported on November 17, 2020 in a 55-year-old man from Wuhan, Hubei, China, and then from China to Iran and then to the rest of the world. 1 On January 30, 2020, World Health Organization (WHO) declared the state of emergency and epidemic and changed the status to the pandemic by March 11, 2020. 2 Till October 11, 51,800,000 plus people confirmed for novel coronavirus disease 2019 (COVID-19) and 1,280,000 plus death recorded in 195 countries. 3 4 In early April 2020, Miller et al announced that countries with Bacillus Calmette-Guérin (BCG) vaccination policies showed a better recovery rate of confirmed cases than countries without compulsory BCG vaccination like Italy, the United States, and the Netherlands. These data suggest that the BCG vaccine shall consider as a potential new prevention-cum-treatment option against COVID-19. 5

## Methodology

For this concise update on COVID-19 disease, we tried to cover all the areas from the genetic point of view to epidemiology, vaccine development, and advanced molecular techniques, like CRISPR/Cas13, either in treatment or diagnosis.

### Origin and Etiology

Based on genomics criteria, coronaviruses (CoVs) have been categorized into three subdivisions: α, β, and γ. The α-CoV group includes species such as porcine epidemic diarrhea coronavirus (PEDV), feline coronavirus (FeCoV), and human coronavirus 229E (HCoV-229E); the β-CoV group includes species like human coronavirus HKU1 (HCoV-HKU1), Middle East respiratory syndrome-coronavirus (MERS-CoV), severe acute respiratory syndrome-coronavirus (SARS-CoV), and bat coronavirus (BCoV); and finally γ-CoV includes species such as Turkey coronavirus (TCoV) and avian infectious bronchitis virus (IBV). 6 The COVID-19, having a + ssRNA genome, belongs to the *Betacoronavirus* group. 6 Phylogenic analysis disclosed that COVID-19 has 90% homology to bat, SL coVZC45 and Bat-SL-CoVZXC21, and had approximately 80 and 50% homology to SARS-CoV and MERS-CoV, respectively. 7

The proteins produced by this virus are short and ranging from 76 to 109 amino acids with molecular weights ranging between 8.4 and 12 kDa. Both protein structures (primary and secondary) elucidate that envelope protein (E) has hydrophilic amino terminus including 7 to 12 amino acids, followed by 25 amino acids' chain and a huge hydrophobic transmembrane domain (TMD) that is ended by a lengthy hydrophilic carboxyl terminus. This region of TMD consists of a minimum of an amphipathic α-helix which oligomerizes to produce a pore in the membrane. This virus contains 29,891 nucleotides which encode for 9,860 amino acids. 8 To add to adenovirus, human metapneumovirus (HmPV), common cold (rhinovirus), human bocavirus, seasonal influenza, parainfluenza virus, this novel coronavirus will be our guest from next winter on. Besides, the above viruses shall express side by side of community-acquired pneumonia (CAP). By conventional molecular techniques, understanding the role of viruses in the setting of pneumonia has nailed a great triumph in the sense of advancement. COVID-19 may cause acute respiratory distress syndrome (ARDS) and spread around the world much severe than SARS, MERS, or even Ebola. 6 Analysis of the COVID-19 RNA genome obtained from confirmed cases from China, Australia, and the United States elucidated that this virus has different mutations. The genomic analysis suggested that these mutated variants of coronavirus (COVID-19) can run-off from antiviral trap sets. This challenge is one of the major setbacks in developing an effective yet safe drug against COVID-19 and even en route to developing its vaccine.

### Clinical Features

The signs and symptoms of coronavirus disease vary from person to person, in a family of three positive cases, one person may be asymptomatic and the others are paucisymptomatic. The disease symptoms include pyrexia, pertussis, dyspnea, malaise, and in severe cases can be multiple organ dysfunction syndromes (MODS), septic shock, and ARDS . CT of the chest demonstrates pneumonia with quiet abnormal reports in all cases. One-third of cases (31%) need to be admitted to ICU and 15% of cases reported to be fatal cases. 9

### Diagnosis

Who is coronavirus patient/suspect? The individual with pyrexia, pharyngitis, or pertussis with a trip to China or other countries of coronavirus hotspot like Italy, Spain, Iran, the United Kingdom, the United States, etc. Nevertheless, cases can be asymptomatic or with no pyrexia. A suspect with the above symptoms becomes confirmed case after a positive kit report. Usually, the molecular test kit is performed on respiratory samples. Coronavirus shall be seen in blood samples in severe cases. Great advances made by the Roche diagnostics, Thermo Fishers Scientific, and Qiagen who came out with multiplex reverse transcription polymerase chain reaction (RT-PCR). 10 11 12

### Differential Diagnosis

Differential diagnostics must encompass the wide range of infectious and noninfectious common respiratory disorders, such as adenovirus, influenza, HmPV, parainfluenza, respiratory syncytial virus (RSV), and rhinovirus. For suspected individuals, other confirmatory tests, such as antigen detection, should be adopted. 13

### Treatment

To date, no cure has been reported for COVID-19. There are numerous reports on potential medicines such as remdesivir that belongs to the class of nucleotide analogs drugs; lopinavir–ritonavir belongs to the protease inhibitor class; oseltamivir belongs to neuraminidase inhibitors; umifenovir belongs to the small molecules class (but approved only in Russia and China and could not pass FDA checkpoints); lamivudine, disoproxil, and tenofovir belong to DNA synthesis inhibitors; and hydroxyl chloroquine and chloroquine. Some alternative medicines from china also proposed but their efficacy so far has not been evaluated including Lianhuaqingwen and Shu Feng Jie du 14 Additionally, 3CLpro-1 (3CLpro inhibitor) and a new vinyl sulfone protease inhibitor have demonstrated great ability to be a powerful antiviral drug against COVID-19. 15 The latest report on clinical trials showing that a team from Monash University, Melbourne, Australia, led by Dr. Kylie Wagstaff, announced that an old and effective antiparasite medicine, named ivermectin, already showed a promising effect in preliminary stages and shall kill the virus within 48 hours. This drug was discovered in 1975 and marketed worldwide by 1981 and shown significant efficacy among in vitro studies against influenza, HIV, zika, and dengue viruses. One of the plus points of this drug is its pricing which is around $0.12 for a complete course of treatment. 16 In the latest try to combat COVID-19, prof. Arturo Casadevall from Johns Hopkins School of Medicine proposed that the well-established method of isolation of plasma, which is a gold standard in blood transfusion, shall be helpful for health care workers primarily, as they are in greater danger as coming in direct interaction with CoV patients. The use of blood-plasma therapy or convalescent plasma technique in cases of COVID-19 obtained an FDA approval. 17 On April 4, 2020, a team of scientists, leads by Vanessa Monteil from Sweden, announced the successful in vitro trial of hrsACE2 with no toxicity and remarkably reduction of COVID-19 infection. 18

## Advances in the Prevention/Treatment of the COVID-19

### Vaccines at Phase-III Clinical Trial

Currently, the Coalition for Epidemic Preparedness Innovations' (CEPI's) candidates from companies, such as Inovio, CureVac, Moderna, AstraZeneca/University of Oxford, Institut Pasteur/Merck/Themis, Clover Biopharmaceuticals, Novavax, University of Queensland/CSL, and the University of Hong Kong, are part of the COVAX initiative. 19 Single-dose Johnson & Johnson vaccine also spread signs of hope around the world. Israel Institute for Biological Research (IIBR) with the scientific cooperation of the MIGAL Galilee Research Institute took the first step toward in vivo trial on a rodent which was reported on April 2, 2020; and in their latest update they proudly announced that the first oral coronavirus will be ready in 90 days for clinical trial. 20 21 . Khodavirdipour et al, in the latest comprehensive paper, reviewed all vaccines in clinical phase-II and -III trails and stated that “they belong to different families of vaccines such as non-replicating viral vector, RNA-based, inactivated form of virus, and DNA-based.” 22

### CRISPR/Cas13 as a Treatment Tool

Analysis of the COVID-19 RNA genome from 19 confirmed cases from China, Australia, and the United States elucidates that this virus has different mutations. They report that these mutations are single nucleotide polymorphism (SNPs), for example, most importantly in open reading frame (ORF8) on amino acids 62 and 84 of COVID-19. These findings complicate the previous idea on the transmission of the virus from bat to man. The genomic analysis suggests that these mutated variants of COVID-19 can run-off from the antiviral trap set in the form of medicines. This challenge is one of the major setbacks in developing an effective yet safe drug against COVID-19 and even en route to developing its vaccine. The result outcomes suggest that this virus obtaining new mutation makes it capable to get away conventional or recent antiviral medicines. The same problems are faced in cases like MERS or SARS and other RNA viruses. Nguyen and colleagues from Beth Israel Deaconess Medical Center, Boston, United States, executed an efficient and flexible approach to target RNA by utilizing the CRISPR/Cas13d method. Nguyen et al suggested that this system shall grind the COVID-19 RNA genome, consequently narrow coronavirus capability to transmit and reproduce. 23

To interfere functionality of COVID-19, they used guide RNAs (gRNAs) which simultaneously trigger the ORF1ab and the spike protein (replicase/transcriptase gene and the *S* gene, respectively). Their work was based on the CRISPR/Cas13d system. This method is an RNA-targeting RNA-guided CRISPR system which can be adapted to chew up the COVID-19 RNA genome by a protein called Cas13d along with the guide RNAs and spacers. One of the plus points of this method is its pliability regarding gRNA design; unlike DNA-editing which needs any nucleotide base (NGG) motif, cleavage of RNA by Cas13d is independent of adjacent sequences. 23 One of the benefits of considering the CRISPR/Cas13d over other methods is its speed and precision in regard to the design of gRNA for a wide variety of viruses which may scape the conventional therapies.

One of the distinctive characteristics of CRISPR/Cas13d fits needs for a fast and precise gRNA design to aim for a wide variety of viruses that may develop and shall run-off conventional medicines. By enlarge, Nguyen et al designed more than 10,000 gRNAs to exactly match the 10 regions of peptide-coding regions of the COVID-19 RNA genome without any matching with the human transcriptome. Because of the high degree of safety and negligible side effect, adeno-associated virus (AAV) shall serve as a vector to transfer the Cas13d effector to the persons with COVID-19. Cas13d effector is small in size, so its size makes Cas13d perfect for “whole-package” AAV delivery along with gRNA array. Likely, up to three gRNAs, targeting different encoding regions of the RNA genome of COVID-19, shall be stowed in an AAV vector and used it systematically for virus clearance and to prevent resistance formation. Moreover, Cas13d expression shall be directed by promoters, such as tissue-specific, to nail the exact cure on the infected organ. Besides, AAVs have serovars that extremely are specific for the lung, which is the main organ caused a fatality in all expired cases, so consequently shall be used in targeted therapy and delivery by this CRISPR method. Interestingly, this method can be extended to other RNA viruses. Because, this technique is so flexible and straightforward, being adapted for any kind of infection caused by RNA viruses.

More trials were needed to determine the efficacy and safety of CRISPR/Cas13d to eliminate COVID-19 and more in vivo tests required before clinical trials on its therapeutic effect. If getting approved, this technique will gift patients globally with more treatment choice to combat with untreatable diseases, caused by viruses that previously could cause resistance quickly. 23 In line with this claim, Dr. Neville E. Sanjana, from New York Genome Center, announced that her center foresees the RNA-triggering enzyme of Cas13 which will revolutionize the concepts in molecular genetics and biology with approaching to the medical purposes. This team was working on developing a new Cas13-based system for screening purposes especially in mammalian cells.

By enlarge, the team managed to collect data for over 24,000 gRNAs which shall help researchers in gRNA design to prevent astray matches on unwanted sites. A simple human cell expresses more than 100,000 RNAs, so the precise triggering of Cas13 is important for therapeutic purposes and screening applications. In total, this technology has good potential to broaden CRISPR applications in the field of molecular and medical genetics. 24 In line with studies, Cockrell et al created a mouse model for MERS coronavirus by CRISPR/Cas9 technique, being used in in vivo studies. 25

### CRISPR/Cas13 as a Diagnostic Tool

Specific high-sensitivity enzymatic reporter unlocking (SHERLOCK) is a method that was primarily announced in 2017 and, to date, improved momentarily. The most latest and sophisticated version is named SHERLOCK v.2. SHERLOCK uses a mixture of Cas13 enzymes besides Csm6 that shows RNase activity while activated by Cas13 nucleases. The combination of all these nucleases permits precise detection of multiple sequences. This technique can clinically be significant for detection and diagnosis whether an obtained specimen of a patient infected by viral agents or not. For example, to diagnose that the patient has dengue, zika, COVID-19, or any other viral infection. Based on SHERLOCK protocol, it can detect any sort of nucleic acid sequence. 26

## Prevention

Beside official guidelines of wearing a face mask, washing hands, and keep social distancing by WHO and Centers for Disease Control and Prevention (CDC), 22 in the very latest finding, the significant preventing role of vitamin-D against COVID-19 has been observed and confirmed by clinicians, scientists. 27

## Conclusion

The outbreak of COVID-19 has challenged public health, medical services, and the economic infrastructure of the world. Only the time will tell us how this outbreak will change and reshape our life in the future. Researchers are trying to introduce potential therapeutics and prevention toolkits. Some advances have been made by antiviral drugs, but none of them have been approved by the FDA; however, treatment with the convalescent plasma has recently been approved by the FDA. Furthermore, hrsACE2 seems very promising. Finally, innovative therapeutics based on the CRISPR/Cas13d might overcome the challenge of COVID-19.
